# Antarctic and Sub-Antarctic Asteroidea database

**DOI:** 10.3897/zookeys.747.22751

**Published:** 2018-04-02

**Authors:** Camille Moreau, Christopher Mah, Antonio Agüera, Nadia Améziane, David Barnes, Guillaume Crokaert, Marc Eléaume, Huw Griffiths, Charlène Guillaumot, Lenaïg G. Hemery, Anna Jażdżewska, Quentin Jossart, Vladimir Laptikhovsky, Katrin Linse, Kate Neill, Chester Sands, Thomas Saucède, Stefano Schiaparelli, Jacek Siciński, Noémie Vasset, Bruno Danis

**Affiliations:** 1 Marine Biology Lab, CP160/15 Université Libre de Bruxelles (ULB) 50, B-1050 Brussels, Belgium; 2 UMR CNRS 6282 Biogéosciences, Université de Bourgogne Franche-Comté (UBFC) 6 Boulevard Gabriel, F-21000 Dijon, France; 3 Department of Invertebrate Zoology, National Museum of Natural History, Smithsonian Institution, Washington, D.C.; 4 Muséum National d’Histoire Naturelle, Station de Biologie Marine, Place de la Croix, BP 225 9182 Concarneau Cedex; 5 British Antarctic Survey, High Cross, Madingley Rd, Cambridge CB3 0ET, United Kingdom; 6 Muséum National d’Histoire Naturelle, Département Origines, UMR7205 ISYEB MNHN-CNRS-UPMC-EPHE, CP51, 57 rue Cuvier, 75231 Paris Cedex 05, France; 7 University of Lodz, Faculty of Biology and Environmental Protection, Department of Invertebrate Zoology and Hydrobiology, Laboratory of Polar Biology and Oceanobiology. 12/16 Banacha st., 90-237 Lodz, Poland; 8 Marine Biology, Vrije Universiteit Brussel (VUB), Pleinlaan 2, 1050 Brussels, Belgium; 9 Centre for Environment, Fisheries and Aquaculture Science, Pakefield Road, Lowestoft NR33 0HT, United Kingdom; 10 Shallow Marine Surveys Group, P.O. Box 598, Stanley FIQQ 1ZZ, Falkland Islands; 11 National Institute of Water and Atmospheric Research, Coasts and Oceans Centre, 301 Evans Bay Parade, Wellington, New Zealand; 12 Dipartimento di Scienze della Terra, dell’Ambiente e della Vita (DISTAV), Università di Genova, C.so Europa 26, Genova I-16132 Italy; 13 Noémie Vasset has been central in compiling all POKER 2 samples, among other things, while she was at the MNHN. She passed away August 22, 2016

**Keywords:** Antarctic, Asteroidea, presence-only data, Southern Ocean, Sub-Antarctic

## Abstract

The present dataset is a compilation of georeferenced occurrences of asteroids (Echinodermata: Asteroidea) in the Southern Ocean. Occurrence data south of 45°S latitude were mined from various sources together with information regarding the taxonomy, the sampling source and sampling sites when available. Records from 1872 to 2016 were thoroughly checked to ensure the quality of a dataset that reaches a total of 13,840 occurrences from 4,580 unique sampling events. Information regarding the reproductive strategy (brooders vs. broadcasters) of 63 species is also made available. This dataset represents the most exhaustive occurrence database on Antarctic and Sub-Antarctic asteroids.

## Introduction

Mapping and understanding life diversity are major issues for the community of biologists and ecologists who focus on the Southern Ocean (SO). For several years, many initiatives such as the International Polar Year, the Census of Antarctic Marine Life (CAML 2005–2010), the Scientific Committee on Antarctic Research: Marine Biodiversity Information Network (SCAR MarBIN, www.biodiversity.aq) or the Biogeographic Atlas of the Southern Ocean (De Broyer et al. 2014) have also gathered information from distinct and transversal scientific domains to provide new multidisciplinary insights in the study of the SO marine ecosystems, linking biogeographic, phylogeographic, physiological, oceanographic, and biogeochemistry data. Such programs have established the most exhaustive and accurate inventories of scientific data ever, since the first historical researches of James Cook in 1772–1775 in the region, and have provided open source information systems (e.g., Register of Antarctic Marine Species, De Broyer and Danis 2010; Global Biodiversity Information Facility, http://www.gbif.org; Ocean Biogeographic Information System http://www.iobis.org/; Van de Putte et al. 2015, http://www.biodiversity.aq).

This extensive assessment was pursued by major improvements in methodologies and data analyses. Improvement of dataset completeness and resolution facilitates modelling approaches (Gutt et al. 2012) that provide interesting tools to better understand distribution patterns in this poorly documented part of the world.

Among benthic taxonomic groups, Asteroidea (Echinodermata) are well represented in the SO with 12% of the global species richness present in the region (Mah and Blake 2012). Around 300 species (Moreau et al. 2015) were reported at all depths including some potential keystone species in benthic communities (McClintock et al. 1988, 2008). As for many taxonomic groups, adaptations of invertebrates to the polar conditions of the SO environments have been widely reported (Peck 2002, 2016) and have led to unique biological traits and life-strategies as well as high levels of endemism in the region (Chown et al. 2015). In particular, reproductive strategies are diversified in the SO with a distinction between brooding and broadcasting species (Poulin et al. 2002; Pearse et al. 2009). In asteroids, the two distinct reproductive strategies strongly drive species distribution patterns and the biogeography of the class in the SO (Moreau et al. 2017).

The present dataset is a compilation of georeferenced occurrences, at species level, for the whole class Asteroidea in the SO. Records from 1872 to 2016 have been gathered from various open source databases. Data collected during recent and unpublished campaigns were also added including records from literature, reaching a total of 13,840 occurrences from 4,580 unique sampling events. This dataset represents the most exhaustive database on Antarctic and Sub-Antarctic asteroids.

## Project description

**Project title**: Antarctic and Sub-Antarctic Asteroidea database

**Personnel**: Camille Moreau, Charlène Guillaumot, Quentin Jossart, Antonio Agüera, Guillaume Crokaert, Marc Eléaume, Thomas Saucède, Katrin Linse, Huw Griffiths, Chester Sands, David Barnes, Vladimir Laptikhovsky, Anna Jażdżewska, Jacek Siciński, Noémie Vasset, Lenaïg G. Hemery, Christopher Mah, Nadia Améziane, Stefano.Schiaparelli, Bruno Danis

**Funding**: The work was supported by a “Fonds pour la formation à la Recherche dans l’Industrie et l’Agriculture” (FRIA) grants to C. Moreau. This is contribution no. 16 to the vERSO project (http://www.versoproject.be), funded by the Belgian Science Policy Office (BELSPO, contract n°BR/132/A1/vERSO). This is contribution to the IPEV programs n°1124 REVOLTA and n°1044 PROTEKER and to team SAMBA of the Biogeosciences laboratory.

**Study area descriptions / descriptor**: This study focuses on the Antarctic and Sub-Antarctic regions located at latitudes south of 45°S. The Southern Ocean is a vast region characterised by the paucity of its scientific data (Griffiths 2010; Griffiths et al. 2011) and available collections are the compilation of several historical campaigns. The objective of this work is to integrate the most complete database of species occurrences for the class Asteroidea in the described geographic extent.

**Design description**: The compilation of occurrence data of asteroid species over the extent of the SO was realised by gathering data available from various biodiversity information systems (OBIS, GBIF, biodiversity.aq, PANGAEA https://www.pangaea.de/) as well as published literature, including original manuscripts (e.g., Gutt et al. 2014; Moles et al. 2015), data papers and cruise reports. Compiled occurrences were complemented with data from personal communications of unpublished works and museums registered collections. This extensive dataset was developed to describe distribution patterns in the SO as well as faunal affinities among 25 Antarctic and Sub-Antarctic bioregions (see Moreau et al. 2017). Several analytical methods such as Bootstrap Spanning Network, non-metrical multidimensional scaling (nMDS) and clustering contributed to highlight the importance of the reproductive strategy on the contemporary observed distribution patterns. The importance of environmental parameters such as influence of Antarctic Circumpolar Current (ACC), the influence of the Polar Front (PF), the presence of gyres or the geographic distance among locations has also been emphasised. This dataset helped to better describe the different biogeographic patterns within asteroids, which are overall congruent with other taxa and differs according to species reproductive strategy. This suggests a differential influence of dispersal capabilities on species distribution patterns. Analyses at genus levels also revealed the underlying legacy of past oceanographic and geodynamic processes in present-day patterns such as the existence of a trans-Antarctic pathway that split the Antarctic continent into two entities in the past. The detailed results are available from Moreau et al. (2017).

**Data description**: Asteroids are common invertebrates of Antarctic benthic communities considering the relative high species richness of the group in the region with regards to the world total diversity (Danis et al. 2014). They play a significant ecological role in Antarctic ecosystems, including in trophic networks (most species being predators) (Dayton 1972; Lawrence 2013). The present dataset, that focuses on regions located at latitudes higher than 45°S, compiles 28 families out of the 39 known worldwide (Mah 2017) with 13,840 occurrences gathered from various sources. The time coverage of the collection starts in 1872 with the HMS Challenger expedition and ends in 2016 with sampled collected during the *RRS James Clark Ross* JR15005 SO-AntEco cruise.

Associated to occurrence data, depth, relative position to the PF, taxonomic information and bioregion were implemented when available. Depth data were extracted from www.gebco.net. Information regarding the reproductive strategy (brooding or broadcasting) of 63 species out of the 299 described was included in the database. Corresponding bioregions of the observed occurrences were specified following Moreau et al. 2017. A significant part of the specimens is deposited in various institutions: e.g., National Museum of Natural History (NMNH), Museum national d’Histoire naturelle (MNHN), Museo Nazionale dell’Antartide (MNA), Université Libre de Bruxelles (ULB), Museo Argentino de Ciencias Naturales (MACN), National Institute of Water and Atmospheric Research (NIWA).

**Quality control description**: Data are available at species level. Nomenclature was thoroughly checked using the Taxon Match Tool implemented in the World Register of Marine Species (WoRMS Editorial Board 2016), to delete all potential discrepancies and update the taxonomy determination. All replicates originating from overlapping origins as well as errors regarding the georeferencing, species synonymy, or misspelling were removed. Most of the occurrences additions originating from recent campaigns were identified by Christopher Mah and Camille Moreau.

## Taxonomic coverage

### General taxonomic coverage description

**Figure 1.**
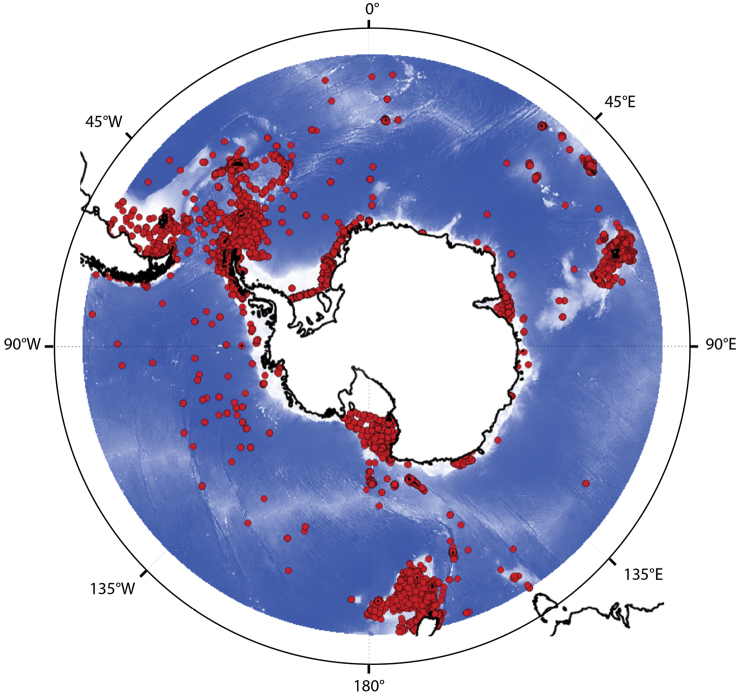
Map of the 13,840 asteroid species occurrences available in the present database, within the boundaries of the Southern Ocean (45°S). Projection: South Pole Stereographic.

The present dataset is the most exhaustive and up-to-date list of available occurrences for the class Asteroidea (Echinodermata), in the entire Southern Ocean. This collection provides information about the occurrence of 28 asteroid families, 118 genera, and 299 species. Occurrence distribution is illustrated on Figure 1.

**Figure 2.**
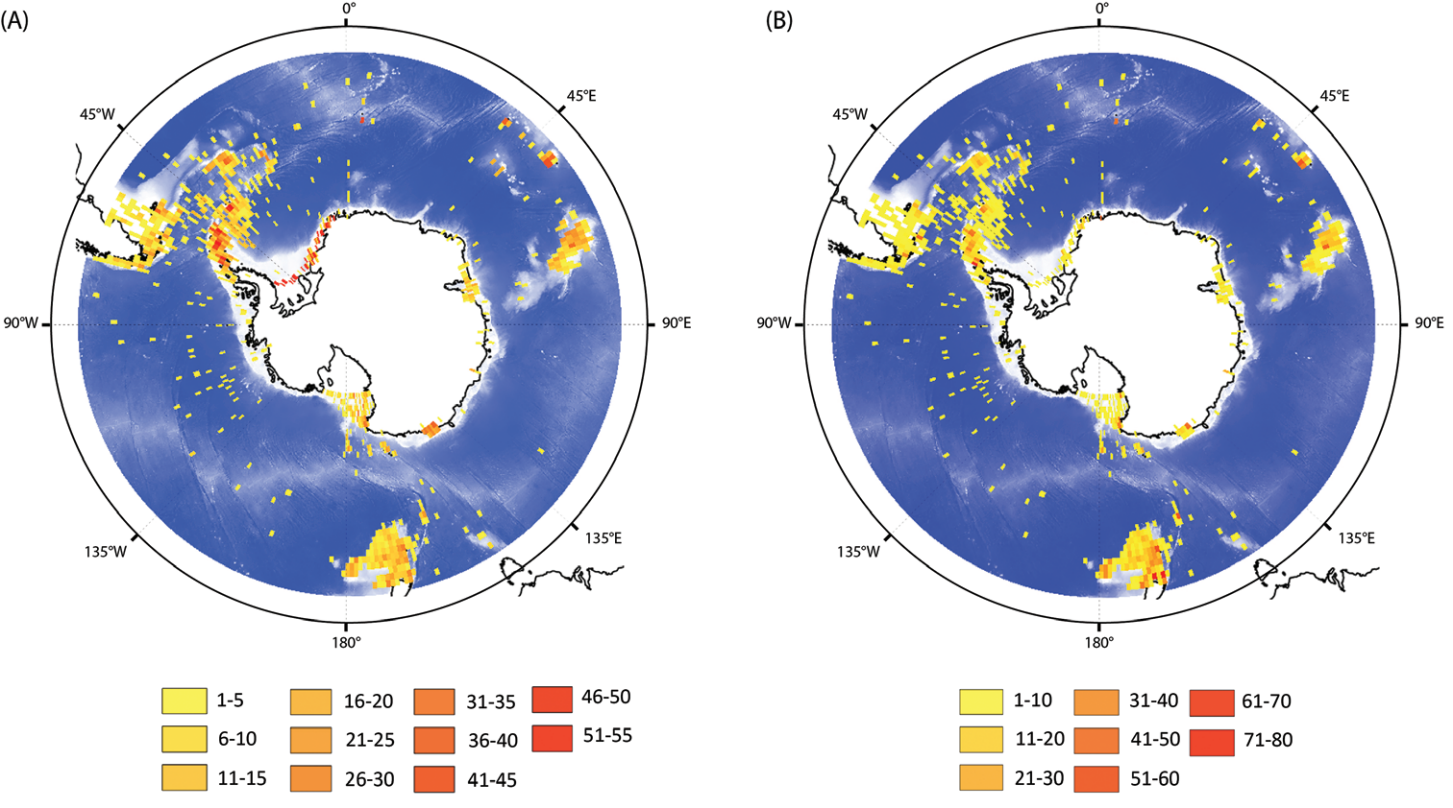
**A** Species richness in the Southern Ocean. The number of asteroid species present in 1° × 1° grid cells are reported using yellow-red colour chart **B** Sampling effort in the Southern Ocean for the class Asteroidea. The number of sampling station per 1° × 1° grid cell is reported using yellow-red colour chart. Projection: South Pole Stereographic.

Species richness in the different regions of the SO was estimated based on 1° × 1° grid cell resolution (Figure 2A). Maximum richness (55 species per cell) was found along the Western Antarctic Peninsula. High richness values were also reported in the Weddell Sea as well as in Sub-Antarctic Islands (Kerguelen, Crozet, Marion, and South Georgia Islands). Richness distribution needs to be interpreted carefully considering the patchy and uneven sampling effort of past oceanographic cruises carried out in the SO (Figure 2B). Indeed, considerable parts of the SO present a crucial lack of sampling. In the context of this study, richness values and sampling effort present a significant positive correlation in space (Pearson r = 0.52, p < 0.001) indicating the need to extend the development of this unique synthesis work and to strengthen the effort for other taxonomic groups.

**Phylum**: Echinodermata

**Class**: Asteroidea

**Order**: Brisingida, Forcipulatida, Notomyotida, Paxillosida, Spinulosida, Valvatida, Velatida

**Family**: Acanthasteridae, Asteriidae, Asterinidae, Astropectinidae, Benthopectinidae, Brisingidae, Ctenodiscidae, Echinasteridae, Freyellidae, Ganeriidae, Goniasteridae, Heliasteridae, Korethrasteridae, Leilasteridae, Luidiidae, Myxasteridae, Odontasteridae, Ophidiasteridae, Paulasteriidae, Pedicellasteridae, Poraniidae, Porcellanasteridae, Pseudarchasteridae, Pterasteridae, Radiasteridae, Solasteridae, Stichasteridae, Zoroasteridae.

**Genus**: *Abyssaster*, *Acanthaster*, *Acodontaster*, *Adelasterias*, *Allostichaster*, *Anasterias*, *Anseropoda*, *Anteliaster*, *Anthenoides*, *Asterina*, *Asthenactis*, *Astromesites*, *Astropecten*, *Astrostole*, *Bathybiaster*, *Belgicella*, *Benthopecten*, *Brisinga*, *Brisingenes*, *Caimanaster*, *Calyptraster*, *Ceramaster*, *Cheiraster*, *Chitonaster*, *Chondraster*, *Cladaster*, *Clavaporania*, *Coscinasterias*, *Cosmasterias*, *Crossaster*, *Cryptasterias*, *Ctenodiscus*, *Cuenotaster*, *Cycethra*, *Diplasterias*, *Diplodontias*, *Diplopteraster*, *Dipsacaster*, *Dytaster*, *Echinaster*, *Eratosaster*, *Eremicaster*, *Freyastera*, *Freyella*, *Freyellaster*, *Fromia*, *Ganeria*, *Gaussaster*, *Gilbertaster*, *Glabraster*, *Granaster*, *Henricia*, *Hippasteria*, *Hymenaster*, *Hymenodiscus*, *Hyphalaster*, *Kampylaster*, *Kenrickaster*, *Labidiaster*, *Leptychaster*, *Lethasterias*, *Lithosoma*, *Lonchotaster*, *Lophaster*, *Luidia*, *Lysasterias*, *Macroptychaster*, *Mediaster*, *Meridiastra*, *Mimastrella*, *Mirastrella*, *Myxoderma*, *Neosmilaster*, *Notasterias*, *Notioceramus*, *Novodinia*, *Odinella*, *Odontaster*, *Odontohenricia*, *Ophidiaster*, *Paralophaster*, *Paranepanthia*, *Patiriella*, *Paulasterias*, *Pectinaster*, *Pedicellaster*, *Pentagonaster*, *Pergamaster*, *Peribolaster*, *Perissasterias*, *Perknaster*, *Persephonaster*, *Pillsburiaster*, *Plutonaster*, *Poraniopsis*, *Porcellanaster*, *Proserpinaster*, *Psalidaster*, *Pseudarchaster*, *Pseudechinaster*, *Psilaster*, *Pteraster*, *Radiaster*, *Remaster*, *Rhopiella*, *Saliasterias*, *Sclerasterias*, *Scotiaster*, *Smilasterias*, *Solaster*, *Sphaeriodiscus*, *Stichaster*, *Styracaster*, *Taranuiaster*, *Tarsaster*, *Tremaster*, *Vemaster*, *Zoroaster*.

**Species**: Abyssaster diadematus, *Abyssaster planus*, *Acanthaster planci*, *Acodontaster capitatus*, *Acodontaster conspicuus*, *Acodontaster elongatus*, *Acodontaster hodgsoni*, *Acodontaster marginatus*, *Adelasterias papillosa*, *Allostichaster capensis*, *Allostichaster farquhari*, *Allostichaster insignis*, *Allostichaster polyplax*, *Anasterias antarctica*, *Anasterias asterinoides*, *Anasterias directa*, *Anasterias laevigata*, *Anasterias mawsoni*, *Anasterias pedicellaris*, *Anasterias perrieri*, *Anasterias rupicola*, *Anasterias sphoerulata*, *Anasterias spirabilis*, *Anasterias studeri*, *Anasterias suteri*, *Anseropoda antarctica*, *Anteliaster australis*, *Anteliaster scaber*, *Anthenoides cristatus*, *Asterina fimbriata*, *Asthenactis australis*, *Astromesites primigenius*, *Astropecten brasiliensis*, *Astrostole scabra*, *Bathybiaster loripes*, *Belgicella racowitzana*, *Benthopecten munidae*, *Benthopecten pedicifer*, *Benthopecten pikei*, *Brisinga chathamica*, *Brisingenes multicostata*, *Caimanaster acutus*, *Calyptraster tenuissimus*, *Calyptraster vitreus*, *Ceramaster australis*, *Ceramaster grenadensis*, *Ceramaster patagonicus*, Cheiraster (Cheiraster) otagoensis, Cheiraster (Luidiaster) antarcticus, Cheiraster (Luidiaster) gerlachei, Cheiraster (Luidiaster) hirsutus, Cheiraster (Luidiaster) planeta, *Chitonaster cataphractus*, *Chitonaster felli*, *Chitonaster johannae*, *Chitonaster trangae*, *Chondraster elattosis*, *Cladaster analogus*, *Clavaporania fitchorum*, *Coscinasterias calamaria*, *Coscinasterias muricata*, *Cosmasterias dyscrita*, *Cosmasterias lurida*, *Crossaster campbellicus*, *Crossaster multispinus*, *Crossaster penicillatus*, *Cryptasterias brachiata*, *Cryptasterias turqueti*, *Ctenodiscus australis*, *Ctenodiscus procurator*, *Cuenotaster involutus*, *Cycethra frigida*, *Cycethra macquariensis*, *Cycethra verrucosa*, *Diplasterias brandti*, *Diplasterias brucei*, *Diplasterias kerguelenensis*, *Diplasterias meridionalis*, *Diplasterias octoradiata*, *Diplasterias radiata*, *Diplodontias dilatatus*, *Diplodontias robustus*, *Diplodontias singularis*, *Diplopteraster clarki*, *Diplopteraster hurleyi*, *Diplopteraster otagoensis*, *Diplopteraster peregrinator*, *Diplopteraster semireticulatus*, *Diplopteraster verrucosus*, *Dipsacaster magnificus*, *Dytaster felix*, *Echinaster farquhari*, *Echinaster smithi*, *Eratosaster jenae*, *Eremicaster crassus*, *Eremicaster pacificus*, *Eremicaster vicinus*, *Freyastera benthophila*, *Freyastera tuberculata*, *Freyella attenuata*, *Freyella drygalskii*, *Freyella echinata*, *Freyella formosa*, *Freyella fragilissima*, *Freyella giardi*, *Freyella heroina*, *Freyella mutabilia*, *Freyellaster polycnema*, *Fromia monilis*, *Ganeria attenuata*, *Ganeria falklandica*, *Ganeria hahni*, *Gaussaster antarcticus*, *Gilbertaster anacanthus*, *Glabraster antarctica*, *Granaster nutrix*, *Henricia aucklandiae*, *Henricia compacta*, *Henricia diffidens*, *Henricia fisheri*, *Henricia lukinsii*, *Henricia obesa*, *Henricia ornata*, *Henricia pagenstecheri*, *Henricia parva*, *Henricia praestans*, *Henricia ralphae*, *Henricia simplex*, *Henricia smilax*, *Henricia spinulfera*, *Henricia studeri*, *Hippasteria falklandica*, *Hippasteria phrygiana*, *Hymenaster caelatus*, *Hymenaster campanulatus*, *Hymenaster carnosus*, *Hymenaster coccinatus*, *Hymenaster crucifer*, *Hymenaster densus*, *Hymenaster edax*, *Hymenaster estcourti*, *Hymenaster formosus*, *Hymenaster fucatus*, *Hymenaster graniferus*, *Hymenaster latebrosus*, *Hymenaster nobilis*, *Hymenaster pellucidus*, *Hymenaster perspicuus*, *Hymenaster praecoquis*, *Hymenaster pullatus*, *Hymenaster sacculatus*, *Hymenodiscus aotearoa*, *Hymenodiscus distincta*, *Hymenodiscus submembranacea*, *Hyphalaster giganteus*, *Hyphalaster inermis*, *Hyphalaster scotiae*, *Kampylaster incurvatus*, *Kenrickaster pedicellaris*, *Labidiaster annulatus*, *Labidiaster radiosus*, *Leptychaster flexuosus*, *Leptychaster kerguelenensis*, *Leptychaster magnificus*, *Leptychaster melchiorensis*, *Lethasterias australis*, *Lithosoma novaezelandiae*, *Lonchotaster tartareus*, *Lophaster densus*, *Lophaster gaini*, *Lophaster stellans*, *Lophaster tenuis*, *Luidia clathrata*, *Luidia porteri*, *Lysasterias adeliae*, *Lysasterias belgicae*, *Lysasterias chirophora*, *Lysasterias digitata*, *Lysasterias hemiora*, *Lysasterias heteractis*, *Lysasterias joffrei*, *Lysasterias lactea*, *Lysasterias perrieri*, *Macroptychaster accrescens*, *Mediaster arcuatus*, *Mediaster dawsoni*, *Mediaster pedicellaris*, *Mediaster sladeni*, *Meridiastra medius*, *Meridiastra oriens*, *Mimastrella cognata*, *Mirastrella biradialis*, *Myxoderma qawashqari*, *Neosmilaster georgianus*, *Neosmilaster steineni*, *Notasterias armata*, *Notasterias bongraini*, *Notasterias candicans*, *Notasterias haswelli*, *Notasterias pedicellaris*, *Notasterias stolophora*, *Notioceramus anomalus*, *Novodinia novaezelandiae*, *Odinella nutrix*, *Odontaster aucklandensis*, *Odontaster benhami*, *Odontaster meridionalis*, *Odontaster pearsei*, *Odontaster penicillatus*, *Odontaster pusillus*, *Odontaster roseus*, *Odontaster validus*, *Odontohenricia anarea*, *Odontohenricia endeavouri*, *Ophidiaster confertus*, *Paralophaster antarcticus*, *Paralophaster godfroyi*, *Paralophaster hyalinus*, *Paralophaster lorioli*, *Paranepanthia aucklandensis*, *Patiriella regularis*, *Paulasterias tyleri*, *Pectinaster filholi*, *Pectinaster mimicus*, *Pedicellaster hypernotius*, *Pentagonaster pulchellus*, *Pergamaster incertus*, *Pergamaster triseriatus*, *Peribolaster folliculatus*, *Peribolaster lictor*, *Peribolaster macleani*, *Perissasterias monacantha*, *Perknaster antarcticus*, *Perknaster aurantiacus*, *Perknaster aurorae*, *Perknaster charcoti*, *Perknaster densus*, *Perknaster fuscus*, *Perknaster sladeni*, *Persephonaster facetus*, *Pillsburiaster aoteanus*, *Pillsburiaster indutilis*, *Plutonaster complexus*, *Plutonaster fragilis*, *Plutonaster hikurangi*, *Plutonaster jonathani*, *Plutonaster knoxi*, *Plutonaster sirius*, *Poraniopsis echinaster*, *Porcellanaster ceruleus*, *Proserpinaster neozelanicus*, *Psalidaster fisheri*, *Psalidaster mordax*, *Pseudarchaster discus*, *Pseudarchaster garricki*, *Pseudechinaster rubens*, *Psilaster acuminatus*, *Psilaster charcoti*, *Pteraster affinis*, *Pteraster bathami*, *Pteraster florifer*, *Pteraster gibber*, *Pteraster hirsutus*, *Pteraster koehleri*, *Pteraster robertsoni*, *Pteraster rugatus*, *Pteraster spinosissimus*, *Pteraster stellifer*, *Radiaster gracilis*, *Remaster gourdoni*, *Rhopiella hirsuta*, *Saliasterias brachiata*, *Sclerasterias eustyla*, *Sclerasterias mollis*, *Scotiaster inornatus*, *Smilasterias clarkailsa*, *Smilasterias irregularis*, *Smilasterias scalprifera*, *Smilasterias triremis*, *Solaster longoi*, *Solaster notophrynus*, *Solaster regularis*, *Solaster torulatus*, *Sphaeriodiscus mirabilis*, *Stichaster australis*, *Styracaster armatus*, *Styracaster chuni*, *Styracaster horridus*, *Styracaster robustus*, *Taranuiaster novaezealandiae*, *Tarsaster stoichodes*, *Tremaster mirabilis*, *Vemaster sudatlanticus*, *Zoroaster actinocles*, *Zoroaster alternicanthus*, *Zoroaster fulgens*, *Zoroaster macracantha*, *Zoroaster spinulosus*, *Zoroaster tenuis*.

**Spatial coverage**: Southern Ocean: from 45°S to higher latitudes

**Temporal coverage**: 1872: HMS Challenger to 2016: JR15005.

**Dataset**: Asteroid occurrences available in the Southern Ocean from 1872 to 2016, collected during different campaigns and gathered from different deposit resources.

**Object name**: Antarctic and Sub-Antarctic Asteroidea Database

**Character encoding**: UTF/8

**Format name**: Darwin Core Archive Format

**Format version**: 1.4

**Distribution**: http://ipt.biodiversity.aq/resource?r=asteroidea_southern_ocean

**Publication date of data**:

**Language**: English

**Metadata language**: English

**Date of metadata creation**:

**Hierarchy level**: Dataset

## References for the dataset

Arnaud P (1964) Echinodermes littoraux de Terre Adelie (Holothuries exceptées) et Pélécypodes commensaux d’Echinides antarctiques 258.

Barnes DKA, Brockington S (2003) Zoobenthic biodiversity, biomass and abundance at Adelaide Island, Antarctica. Marine Ecology Progress Series 249: 145–55. https://doi.org/10.3354/meps249145

Beckley LE, Branch GM (1992) A quantitative scuba-diving survey of the sublittoral macrobenthos at subantarctic Marion Island. Polar Biology 11(8): 553–563. https://doi.org/10.1007/BF00237948

Belman BW, Giese AC (1974) Oxygen consumption of an asteroid and an echinoid from the Antarctic. The Biological Bulletin 146(2): 157–164. https://doi.org/10.2307/1540614

Benham WB (1909) The echinoderms, other than holothurians, of the Subantarctic islands of New Zealand, 295–305.

Blankley WO, Branch GM (1984) Co-operative prey capture and unusual brooding habits of *Anasterias rupicola* (Verrill)(Asteroidea) at sub-Antarctic Marion Island. Marine Ecology Progress Series 20(1/2): 171–176. https://doi.org/10.3354/meps020171

Bosch I, Pearse JS (1990) Developmental types of shallow-water asteroids of McMurdo Sound, Antarctica. Marine Biology 104(1): 41–46. https://doi.org/10.1007/BF01313155

Bosch I, Slattery M (1999) Costs of extended brood protection in the Antarctic sea star, *Neosmilaster georgianus* (Echinodermata: Asteroidea). Marine Biology 134(3): 449–459. https://doi.org/10.1007/s002270050561

Cherbonnier G (1974) Invertébrés de l’infralittoral rocheux dans l’Archipel de Kerguelen. Holothurides et Échinides. Le fascicule 35. Comité national français des recherches antarctiques 3: 27–31.

Clark AM (1962) Asteroidea. *B.A.N.Z*. Antarctic Research Expedition 1929–1931. B9: 1–104.

Conlan KE, Rau GH, Kvitek RG (2006) δ13C and δ15N shifts in benthic invertebrates exposed to sewage from McMurdo Station, Antarctica. Marine Pollution Bulletin 52(12): 1695–1707. https://doi.org/10.1016/j.marpolbul.2006.06.010

Cranmer TL, Ruhl HA, Baldwin RJ, Kaufmann RS (2003) Spatial and temporal variation in the abundance, distribution and population structure of epibenthic megafauna in Port Foster, Deception Island. Deep Sea Research Part II: Topical Studies in Oceanography 50(10): 1821–1842. https://doi.org/10.1016/S0967-0645(03)00093-6

Dayton PK, Robilliard GA, Paine RT (1970) Benthic faunal zonation as a result of anchor ice at McMurdo Sound, Antarctica. In: Holgate MW (Ed.) Antarctic ecology Academic Press, London, 244–258.

Dayton PK (1972) Toward an understanding of community resilience and the potential effects of enrichments to the benthos at McMurdo Sound, Antarctica. Proceedings of the colloquium on conservation problems in Antarctica Allen Press Lawrence, Kansas, 81–96.

Dearborn JH, Edwards KC, Fratt DB (1991) Diet, feeding behavior, and surface morphology of the multi-armed Antarctic sea star *Labidiaster annulatus* (Echinodermata: Asteroidea). Marine Ecology Progress Series 77(1): 65–84. https://doi.org/10.3354/meps077065

De Moreno JEA, Gerpe MS, Moreno VJ, Vodopivez C (1997) Heavy metals in Antarctic organisms. Polar Biology 17(2): 131–140. https://doi.org/10.1007/s003000050115

Desbruyères D, Guille A (1973) La Faune benthique de l’Archipel de Kerguelen. Premières données quantitatives. Comptes Rendus de l’Académie des Sciences, Paris 276: 633–636.

Duquesne S, Riddle M (2002) Biological monitoring of heavy-metal contamination in coastal waters off Casey Station, Windmill Islands, East Antarctica. Polar Biology 25(3): 206–215.

Fell HB (1953) Echinoderms from the Subantarctic islands of New Zealand: Asteroidea, Ophiuroidea, and Echinoidea, Records Dominion Museum 2: 73–111.

Fell HB, Clark HE (1959) *Anareaster*, a new genus of Asteroidea from Antarctica. Transactions of the Royal Society of New Zealand 87: 185–187.

Fernanda PA, Clementina BC, Javier C, Gabriela M (2015) Reproduction and oxidative metabolism in the brooding sea star *Anasterias antarctica* (Lütken, 1957). Journal of Experimental Marine Biology and Ecology 463: 150–157. https://doi.org/10.1016/j.jembe.2014.11.009

Gillies CL, Stark JS, Johnstone GJ, Smith SD (2012) Carbon flow and trophic structure of an Antarctic coastal benthic community as determined by δ13C and δ15N. Estuarine, Coastal and Shelf Science 97: 44–57. https://doi.org/10.1016/j.ecss.2011.11.003

Global Biology Information Facility. http://www.gbif.org/ [07/2016]

Grygier MJ (1987) Antarctic records of asteroid-infesting Ascothoracida (Crustacea), including a new genus of Ctenosculidae. Proceedings of the Biological Society of Washington 100 (4): 700–712.

Guille A (1977) Benthic bionomy of the continental shelf of the Kerguelen Islands: quantitative data on the echinoderms of the Morbihan Gulf. In: Llano GA (Ed.) Adaptations within Antarctic ecosystems Smithsonian Institution Washington, DC, 253–262.

Gutt J, Barratt I, Domack EW, d’Udekem d’Acoz C, Dimmler W, Grémare A, Heilmayer O, Isla E, Janussen D, Jorgensen E, Kock KH, Lehnert LS, López-Gonzáles PJ, Langner S, Linse K, Manjón-Cabeza ME, Meißner M, Montiel A, Raes M, Robert H, Rose A, Schepisi ES, Saucède T, Scheidat M, Schenke HW, Seiler J, Smith C (2010) Macrobenthos in surface sediments sampled during POLARSTERN cruise ANT-XXIII/8. https://doi.org/10.1594/PANGAEA.718106, In supplement to: Gutt J et al. (2011): Biodiversity change after climate-induced ice-shelf collapse in the Antarctic. Deep Sea Research Part II: Topical Studies in Oceanography 58(1–2): 74–83. https://doi.org/10.1016/j.dsr2.2010.05.024

Gutt J, Piepenburg D, Voß J (2014) Asteroids, ophiuroids and holothurians from the southeastern Weddell Sea (Southern Ocean). ZooKeys 434: 1–15. https://doi.org/10.3897/zookeys.434.7622

Intergovernmental Oceanographic Commission (IOC) of UNESCO (2015) The Ocean BiogeographicInformation System. http://www.iobis.org

Janosik AM, Mahon AR, Scheltema RS, Halanych KM (2008) Short Note: Life history of the Antarctic sea star *Labidiaster annulatus* (Asteroidea: Labidiasteridae) revealed by DNA barcoding. Antarctic Science 20(6): 563–564. https://doi.org/10.1017/S0954102008001533

Janosik AM, Halanych KM (2010) Unrecognized Antarctic biodiversity: a case study of the genus *Odontaster* (Odontasteridae; Asteroidea). Integrative and comparative biology: icq119. https://doi.org/10.1093/icb/icq119

Koehler R (1907) Astéries, Ophiures et Echinides recueillis dans les mers australes par la Scotia (1902–1904). Zoologischer Anzeiger 32(6): 140–147.

Koehler R (1908) Astéries, Ophiures et Echinides de l’Expédition Antarctique Nationale Écossaise. Transactions of the Royal Society of Edinburgh 46: 529–649.

Koehler R (1912) Echinodermes (Asteries, Ophiures et Echinides). Deuxième Expédition Antarctique Francaise 1908–1910 J. Charcot. Masson et cie, Paris, 1–277.

Koehler R (1920) Echinodermata: Asteroidea. Scientific Reports, Australasian Antarctic Expedition (1911–1914) C8: 1–308.

Koehler R (1923) Astéries et Ophiures. Further zoological results of the Swedish Antarctic Expedition (1901–1903) 1: 1–145.

Korovchinsky NM (1976) New species of abyssal starfishes of the genus Freyella (Brisingidae) from the South Atlantic. Zoologicheskii Zhurnal 55(8): 1187–1194.

Lawrence JM (2013). Starfish: biology and ecology of the Asteroidea. John Hopkins University Press, 288 pp.

Lawrence JM, McClintock JB, Guille A (1984) Organic level and caloric content of eggs of brooding asteroids and an echinoid (Echinodermata) from Kerguelen (South Indian Ocean). International Journal of Invertebrate Reproduction and Development 7(4): 249–257.

Lawrence JM, McClintock JB (1987) Intertidal invertebrate and algal communities on the rocky shores of the Bay of Morbihan, Kerguelen (South Indian Ocean). Marine Ecology 8(3): 207–220.

Lovell LL, Trego KD (2003) The epibenthic megafaunal and benthic infaunal invertebrates of Port Foster, Deception Island (South Shetland Islands, Antarctica). Deep Sea Research Part II: Topical Studies in Oceanography 50(10): 1799–1819.

Ludwig H (1903) Seesterne. Résultats du voyage du S.Y. Belgica en 1897-1898-1899. Rapports scientifiques, 1–72.

Mah C, Linse K, Copley J, Marsh L, Rogers A, Clague D, Foltz D (2015) Description of a new family, new genus, and two new species of deep‐sea Forcipulatacea (Asteroidea), including the first known sea star from hydrothermal vent habitats. Zoological Journal of the Linnean Society 174(1): 93–113.

McClintock JB, Pearse JS (1986) Organic and energetic content of eggs and juveniles of Antarctic echinoids and asterids with lecithotrophic development. Comparative Biochemistry and Physiology Part A: Physiology 85(2): 341–345.

McClintock JB, Baker BJ (1997) Palatability and chemical defense of eggs, embryos and larvae of shallow-water Antarctic marine invertebrates. Marine Ecology Progress Series 154: 121–131.

McClintock JB, Baker BJ, Amsler CD, Barlow TL (2000) Chemotactic tube-foot responses of the spongivorous sea star *Perknaster fuscus* to organic extracts of sponges from McMurdo Sound, Antarctica. Antarctic Science 12(1): 41–46.

McClintock JB, Amsler MO, Amsler CD, Baker BJ (2006) The biochemical composition, energy content, and chemical antifeedant defenses of the common Antarctic Peninsular sea stars *Granaster nutrix* and *Neosmilaster georgianus*. Polar Biology 29(7): 615–623.

McClintock JB, Angus RA, Ho CP, Amsler CD, Baker BJ (2008) Intraspecific agonistic arm-fencing behavior in the Antarctic keystone sea star *Odontaster validus* influences prey acquisition. Marine Ecology Progress Series 371: 297–300.

McKnight DG (2006) The Marine Fauna of New Zealand, Echinodermata: Asteroidea (Sea-stars): Order Velatida, Spinulosida, Forcipulatida, Brisingida, with Addenda to Paxillosida, Valvatida. Biodiversity Memoirs 120 National Institute of Water and Atmospheric Research, 187 pp.

Moles J, Figuerola B, Campanyà-Llovet N, Monleón-Getino T, Taboada S, Avila C (2015) Distribution patterns in Antarctic and Subantarctic echinoderms. Polar Biology 38(6): 799–813.

Pearse JS, Giese AC (1966) The organic constitution of several benthonic invertebrates from McMurdo Sound, Antarctica. Comparative biochemistry and physiology 18(1): 47IN151–50IN257.

POLARSTERN cruise ANT-XXIII/8. https://doi.org/10.1594/PANGAEA.718106, In supplement to: Gutt J et al. (2011) Biodiversity change after climate-induced ice-shelf collapse in the Antarctic. Deep Sea Research Part II: Topical Studies in Oceanography 58(1/2): 74–83. https://doi.org/10.1016/j.dsr2.2010.05.024

Presler P, Figielska E (1997) New data on the Asteroidea of Admiralty Bay, King George Island, South Shetland Islands. Polish Polar Research 18: 107–117.

Shilling FM, Manahan DT (1994) Energy metabolism and amino acid transport during early development of Antarctic and temperate echinoderms. The Biological Bulletin 187(3): 398–407.

Silva JR, Peck L (2000) Induced in vitro phagocytosis of the Antarctic starfish *Odontaster validus* (Koehler 1906) at 0° C. Polar Biology 23(4): 225–230.

Sladen WP (1889) Report on the scientific results of the voyage of *HMS Challenger* during the years 1873–76. Zoology 30.

Sladen WP (1882) The Asteroidea of HMS *Challenger* Expedition. (Preliminary notices). 1. Pterasteridae. Journal of the Linnean Society of London 16: 186–246

Slattery M, Bosch I (1993) Mating behavior of a brooding Antarctic asteroid, *Neosmilaster georgianus*. Invertebrate Reproduction & Development 24(2): 97–102.

Smale DA, Barnes DK, Fraser KP, Mann PJ, Brown MP (2007) Scavenging in Antarctica: intense variation between sites and seasons in shallow benthic necrophagy. Journal of Experimental Marine Biology and Ecology 349(2): 405–417.

Stanwell-Smith D, Clarke A (1998) Seasonality of reproduction in the cushion star *Odontaster validus* at Signy Island, Antarctica. Marine Biology 131(3): 479–487.

Taboada S, Núñez-Pons L, Avila C (2013) Feeding repellence of Antarctic and sub-Antarctic benthic invertebrates against the omnivorous sea star *Odontaster validus*. Polar Biology 36(1): 13–25.

Thomson C (1876) Notice of some Peculiarities in the Mode of Propagation of certain Echinoderms of the Southern Sea. Zoological Journal of the Linnean Society 13(66): 55–79.

Van de Putte A, Youdjou N, Danis B (2015) The Antarctic Biodiversity Information Facility. http://www.biodiversity.aq

Winkler M, Fillinger L, Funke T, Richter C, Laudien J (2013) Succession of benthic hard-bottom communities abundance at station Errina2012_MDD4. https://doi.org/10.1594/PANGAEA.806083

## References regarding the reproduction strategy

Arnaud PM (1974) Contribution à la bionomie marine benthique des régions antarctiques et sub-antarctiques. Tethys 6: 467–653.

Barker MF (1978) Structure of the organs of attachment of brachiolaria larvae of *Stichaster australis* (Verrill) and *Coscinasterias calamaria* (Gray)(Echinodermata: Asteroidea). Journal of Experimental Marine Biology and Ecology 33(1): 1–36.

Bosch I, Pearse JS (1990) Developmental types of shallow-water asteroids of McMurdo Sound, Antarctica. Marine Biology 104(1): 41–46.

Bosch I (1989) Contrasting modes of reproduction in two Antarctic asteroids of the genus *Porania*, with a description of unusual feeding and non-feeding larval types. The Biological Bulletin 177(1): 77–82.

Byrne M (2006) Life history diversity and evolution in the Asterinidae. Integrative and Comparative Biology 46(3): 243–254.

Crump RG (1971) Annual reproductive cycles in three geographically separated populations of *Patiriella regularis* (Verrill), a common New Zealand asteroid. Journal of Experimental Marine Biology and Ecology 7(2): 137–162.

Dehn PF (1982) The effect of food and temperature on reproduction in *Luidia clathrata* (Asteroidea). Echinoderms: proceedings of the international, Tampa Bay. Balkema, Rotterdam, 457–463.

Fisher WK (1940) Asteroidea. ‘Discovery’ Reports 20: 69–306.

Henderson JA, Lucas JS (1971) Larval development and metamorphosis of *Acanthaster planci* (Asteroidea). Nature 232: 655–657.

Hyman LH (1955) The invertebrates: Echinodermata. McGraw-Hill Book Company, New York, 763 pp.

Janosik AM, Mahon AR, Scheltema RS, Halanych KM (2008) Short Note: Life history of the Antarctic sea star *Labidiaster annulatus* (Asteroidea: Labidiasteridae) revealed by DNA barcoding. Antarctic Science 20(6): 563–564.

Lawrence JM, McClintock JB, Guille A (1984) Organic level and caloric content of eggs of brooding asteroids and an echinoid (Echinodermata) from Kerguelen (South Indian Ocean). International Journal of Invertebrate Reproduction and Development 7(4): 249–257.

Lieberkind I (1926) *Ctenodiscus australis* Lütken. A brood-protecting asteroid. Vid. Dansk. Nat. Hist. Foren. 82: 184–196

MacBride EW (1920) Echinodermata (Part II) and Enteropneusta. Larvae of Echinoderma and Enteropneusta. British Antarctic (Terra Nova) Expedition, 1910. Natural History Report, Zoology 4: 83–94.

O’Hara TD (1998) Origin of Macquarie Island echinoderms. Polar Biology 20(2): 143–151.

Pain SL, Tyler PA, Gage JD (1982) The reproductive biology of the deep-sea asteroids *Benthopecten simplex* (Perrier), *Pectinaster filholi* Perrier, and *Pontaster tenuispinus* Düben & Koren (Phanerozonia: Benthopectinidae) from the Rockall Trough. Journal of Experimental Marine Biology and Ecology 65(2): 195–211.

Pearse JS (1965) Reproductive periodicities in several contrasting populations of *Odontaster validus* Koehler, a common Antarctic asteroid. Antarctic Research Series 5: 39–85.

Pearse JS, McClintock JB, Bosch I (1991) Reproduction of Antarctic benthic marine invertebrates: tempos, modes, and timing. American Zoologist 31(1): 65–80.

Pearse JS, Bosch I (1994) Brooding in the Antarctic: Östergren had it nearly right. Echinoderms through time. Balkema, Rotterdam, 111–120.

Simpson RD (1982) The reproduction of some echinoderms from Macquarie Island. Australian Museum Memoirs 16: 39–52.

Strathmann RR (1987) Reproduction and development of marine invertebrates of the northern pacific coast. University of Washington press, Seattle, Washington, 670 pp.

Thorson G (1936) The larval development, growth, and metabolism of arctic marine bottom invertebrates compared with those of other seas. CA Reitzel 100(6).

Tyler PA, Pain SL, Gage JD, Billett DSM (1984) The reproductive biology of deep-sea forcipulate seastars (Asteroidea: Echinodermata) from the NE Atlantic Ocean. Journal of the Marine Biological Association of the United Kingdom 64(3): 587–601.
